# Potential Cross-Transmission of Mycobacterium abscessus among Non-Cystic Fibrosis Patients at a Tertiary Hospital in Japan

**DOI:** 10.1128/spectrum.00097-22

**Published:** 2022-05-10

**Authors:** Keiji Fujiwara, Mitsunori Yoshida, Yoshiro Murase, Akio Aono, Koji Furuuchi, Yoshiaki Tanaka, Ken Ohta, Manabu Ato, Satoshi Mitarai, Kozo Morimoto

**Affiliations:** a Respiratory Disease Center, Fukujuji Hospital, Japan Anti-Tuberculosis Association, Tokyo, Japan; b Department of Mycobacterium Reference and Research, The Research Institute of Tuberculosis, Japan Anti-Tuberculosis Association, Tokyo, Japan; c Department of Basic Mycobacteriosis, Nagasaki University Graduate School of Biomedical Sciences, Nagasaki, Japan; d Department of Mycobacteriology, Leprosy Research Center, National Institute of Infectious Diseases, Tokyo, Japan; e Division of Clinical Research, Fukujuji Hospital, Japan Anti-Tuberculosis Association, Tokyo, Japan; Johns Hopkins University School of Medicine

**Keywords:** nontuberculous mycobacteria, *Mycobacterium abscessus* subsp. *massiliense*, whole-genome sequencing, variable-number tandem repeats, transmission

## Abstract

*Mycobacterium abscessus* (M. abscessus) is a highly antimicrobial-resistant pathogen that causes refractory pulmonary disease. Recently, the possibility of M. abscessus cross-transmission among cystic fibrosis (CF) patients has been reported. CF is rare in Asia, but M. abscessus pulmonary disease is common. Therefore, we investigated the possibility of M. abscessus cross-transmission in a Japanese hospital setting. Of 104 M. abscessus isolates, 25 isolates from 24 patients were classified into four clusters based on their variable number of tandem repeat profiles and were subjected to whole-genome sequencing (WGS). The epidemiological linkages among our patients were investigated by integrating the WGS data of previously reported nosocomial outbreak-related M. abscessus clinical isolates in the United Kingdom and the United States. Eight transmissible clusters (TCs) were identified. The United Kingdom and United States isolates were assigned to four clusters (TC1, TC2, TC5, and TC8) and one cluster (TC3), respectively. A total of 12 isolates from our hospital belonged to 4 clusters (TC4, TC5, TC6, and TC7). Epidemiological linkage analysis inferred direct or indirect transmission between patients in our hospital in TC4 and TC5 but not in TC6 and TC7. In TC5, the single nucleotide polymorphism distance between isolates from Japanese and United Kingdom patients was less than 21; however, there was no contact. This study revealed that genetically closely related isolates exist, even in non-CF patients. However, the transmission route remains unclear, and further research is warranted to clarify whether cross-transmission is involved.

**IMPORTANCE** Although the possibility of *Mycobacterium abscessus* (M. abscessus) cross-transmission in cystic fibrosis (CF) patients has often been reported, it is not clear whether similar events have occurred in Asian non-CF patients. Whole-genome sequencing analysis of M. abscessus isolates from Fukujuji Hospital in Japan indicated that genetically closely related M. abscessus isolates exist. In addition, according to epidemiological linkage analysis, some clusters were suspected of direct or indirect transmission between patients within our hospital. However, the transmission route of M. abscessus remains unclear, because interestingly, one cluster showed a single nucleotide polymorphism distance of less than 21 from the United Kingdom isolates, but no epidemiological linkage was identified.

## INTRODUCTION

The prevalence of nontuberculous mycobacterial (NTM) pulmonary disease has been increasing worldwide in recent decades (1, 2). *Mycobacterium abscessus* (M. abscessus) is a highly antimicrobial-resistant NTM that causes refractory disease (3). Two systematic reviews reported that the treatment success for M. abscessus pulmonary disease was 45.6% to 53.2%, which was completely unsatisfactory (4, 5). In addition to the treatment difficulty, the possibility of cross-transmission of M. abscessus among cystic fibrosis (CF) patients has recently been reported (6–8), indicating the importance of infection control. Thus, it is crucial to identify the transmission routes of M. abscessus to prevent recurrent infections.

In the context of NTM isolate genotyping, various techniques, such as pulsed-field gel electrophoresis, variable-number tandem repeats (VNTR), and multilocus sequence typing, have been utilized (7, 9–11). Recent advances in whole-genome sequencing (WGS) have enabled the detailed bacteriological and epidemiological analysis of NTM species. Bryant et al. reported the potential for cross-transmission of M. abscessus in a CF center in the United Kingdom by analyzing pairwise single nucleotide polymorphism (SNP) distances (6). They defined less than or equal to 25 SNP distances within the conserved genomic region as the threshold for cross-transmission in their sample set. Subsequent studies have generally used similar thresholds (12, 13). However, this SNP distance threshold was determined by a single study, and it is not clear if this threshold can be directly adapted universally.

Global genetic research on 1,080 M. abscessus clinical isolates from 517 CF patients in Europe, the United States, and Australia revealed that approximately 74% of CF patients worldwide were infected with two M. abscessus subsp. *abscessus* clones or one M. abscessus subsp. *massiliense* clone (14). Although the findings suggest the possibility of dominant circulating clones (DCCs) among CF patients, few studies have been conducted using WGS in combination with clinicoepidemiological profiles to investigate whether similar events occur among non-CF patients (15, 16).

A previous multicenter study involving 121 M. abscessus pulmonary disease patients analyzed the relationship between the VNTR profiles and clinical characteristics; however, no apparent relationship was identified (3). On the other hand, a few clusters with identical VNTR profiles were detected, suggesting the cross-transmission of a few clones (12, 17). Accordingly, in the present study, we aimed to clarify the possibility of cross-transmission among non-CF patients by not only performing WGS analysis of M. abscessus forming VNTR clusters but also by investigating epidemiological linkage data.

## RESULTS

### Characteristics of the study patients.

A total of 25 isolates were obtained from the 24 patients enrolled in the present study. Of these, 23 patients met the diagnostic criteria of NTM pulmonary disease established by the American Thoracic Society/European Respiratory Society/European Society of Clinical Microbiology and Infectious Diseases/Infectious Diseases Society of America (ATS/ERS/ESCMID/IDSA), but 1 did not (18). The characteristics of the patients are shown in Table 1. A total of 15 patients were female (62.5%), the median age was 67 years (range, 54 to 71), and 6 (25.0%) had a previous NTM pulmonary disease history (4 patients had Mycobacterium avium complex [MAC] infection, 1 had MAC and Mycobacterium lentiflavum infection, and 1 had Mycobacterium gordonae infection). The most common radiological finding was noncavitary nodular bronchiectatic (NC-NB; 12 patients, 50.0%), followed by cavitary nodular bronchiectatic (C-NB; 6, 25.0%), fibrocavitary (FC; 5, 20.8%), and unclassified type (1, 4.2%). Colony morphotypes were classified as follows: rough, 40% (10 isolates); smooth, 44% (11); mixed, 8% (2); and intermediate, 8% (2). Regarding susceptibility to clarithromycin, all M. abscessus subsp. *abscessus* isolates had inducible resistance, while all but one M. abscessus subsp. *massiliense* isolate was susceptible. All isolates were amikacin-susceptible or intermediate.

**TABLE 1:** Baseline characteristics of the study patients^*a*^

Patient (RGM no.)	Subspecies	VNTR profile	Sequence clade	Sex	Age	Sample date (mo/day/yr)	Colony morphotype	CLR MIC (μg/mL)	AMK MIC (μg/mL)	Respiratory disease(s)	Systemic disease(s)	Radiographic findings	Outcome
174	Mabs	I		F	70	12/29/2011	Smooth	32	32			NC-NB	No treatment
185	Mabs	I		F	69	4/11/2012	Smooth	32	16		DM, UC	C-NB	No treatment
18	Mmas	II		F	72	10/21/2004	Smooth	0.5	32		HF	NC-NB	No treatment
76	Mmas	II		F	58	5/1/2008	Rough	0.25	32	COPD		C-NB	Treatment success by antibiotics and operations
102	Mmas	II		M	63	12/10/2008	Intermediate	0.5	16	COPD, p-TB, Asp	GE disease	FC	Treatment success by antibiotics
103	Mmas	II		F	82	12/16/2008	Rough	0.25	8	BA, p-NTM		NC-NB	No treatment
118	Mmas	II	SC4	F	71	9/7/2009	Smooth	0.5	32	LC	BC	NC-NB	Treatment success by antibiotics
162	Mmas	II	SC4	F	44	8/10/2011	Smooth	0.5	32	p-TB		NC-NB	Treatment success by antibiotics
176	Mmas	II		F	54	1/24/2012	Smooth	32	32	p-NTM	Depression	NC-NB	Treatment by antibiotics, but no success
239	Mmas	II	SC4	F	54	12/12/2013	Smooth	0.25	32	p-NTM		NC-NB	Treatment success by antibiotics
249	Mmas	II		M	68	6/6/2014	Smooth	0.5	16	p-NTM	GE disease	C-NB	Treatment success by antibiotics
255	Mmas	II		M	73	8/12/2014	Rough	0.12	32			C-NB	Treatment success by antibiotics
3	Mmas	III		M	53	2/18/2004	Mixed	0.5	32		GE disease	FC	Treatment success by antibiotics and operations
105	Mmas	III	SC7	F	78	1/10/2009	Smooth	0.25	16	p-TB	DM, CV disease, GE disease	NC-NB	No treatment
119	Mmas	III		M	51	9/16/2009	Rough	0.12	32	p-TB		FC	Treatment success by antibiotics
129	Mmas	III		F	74	4/27/2010	Mixed	0.5	32	p-NTM, TB pleurisy	GE disease, BC	NC-NB	No treatment
156	Mmas	III	SC6	M	61	4/23/2011	Rough	0.25	32	p-TB	DM, CL disease	FC	No treatment
158	Mmas	III	SC6	F	67	7/5/2011	Smooth	0.12	16	p-NTM	Sinusitis	C-NB	Treatment success by antibiotics
178	Mmas	III	SC6	M	55	2/1/2012	Rough	0.25	32	p-TB		NC-NB	No treatment
223	Mmas	III		M	77	8/12/2013	Smooth	0.25	16	COPD, a-TB		Unclassified	No treatment
253	Mmas	III	SC7	F	52	7/10/2014	Rough	0.06	2		HT	C-NB	Treatment success by antibiotics
19	Mmas	IV	SC5	M	66	12/20/2004	Intermediate	1	8	IP, COPD, BA, p-TB	DM	NC-NB	No treatment
44	Mmas	IV	SC5	F	67	7/26/2006	Rough	0.25	16			NC-NB	Treatment by antibiotics, but no success
96 (pre)	Mmas	IV	SC5	F	48	10/28/2008	Rough	1	8			FC	Treatment success by antibiotics and operations
96 (post)	Mmas		SC5		52	9/24/2012	Rough	1	8				

a Mabs, Mycobacterium abscessus subsp. *abscessus*; Mmas. Mycobacterium abscessus subsp. *massiliense*; VNTR, variable-number tandem repeats; SC, sequence clade; M, male; F, female; CLR, clarithromycin; AMK, amikacin; COPD, chronic obstructive pulmonary disease; p-TB, previous tuberculosis; Asp, aspergillosis; BA, bronchial asthma; p-NTM, previous nontuberculous mycobacteria; LC, lung cancer; TB, tuberculosis; a-TB, active tuberculosis; IP, interstitial pneumonia; DM, diabetes mellitus; UC, uterine cancer; HF, heart failure; GE, gastroesophageal; BC, breast cancer; CV, cerebrovascular; CL, chronic liver; HT, hyperthyroidism; NC-NB, noncavitary nodular bronchiectatic; C-NB, cavitary nodular bronchiectatic; FC, fibrocavitary.

### Phylogenetic analysis results.

**FIG 1:**
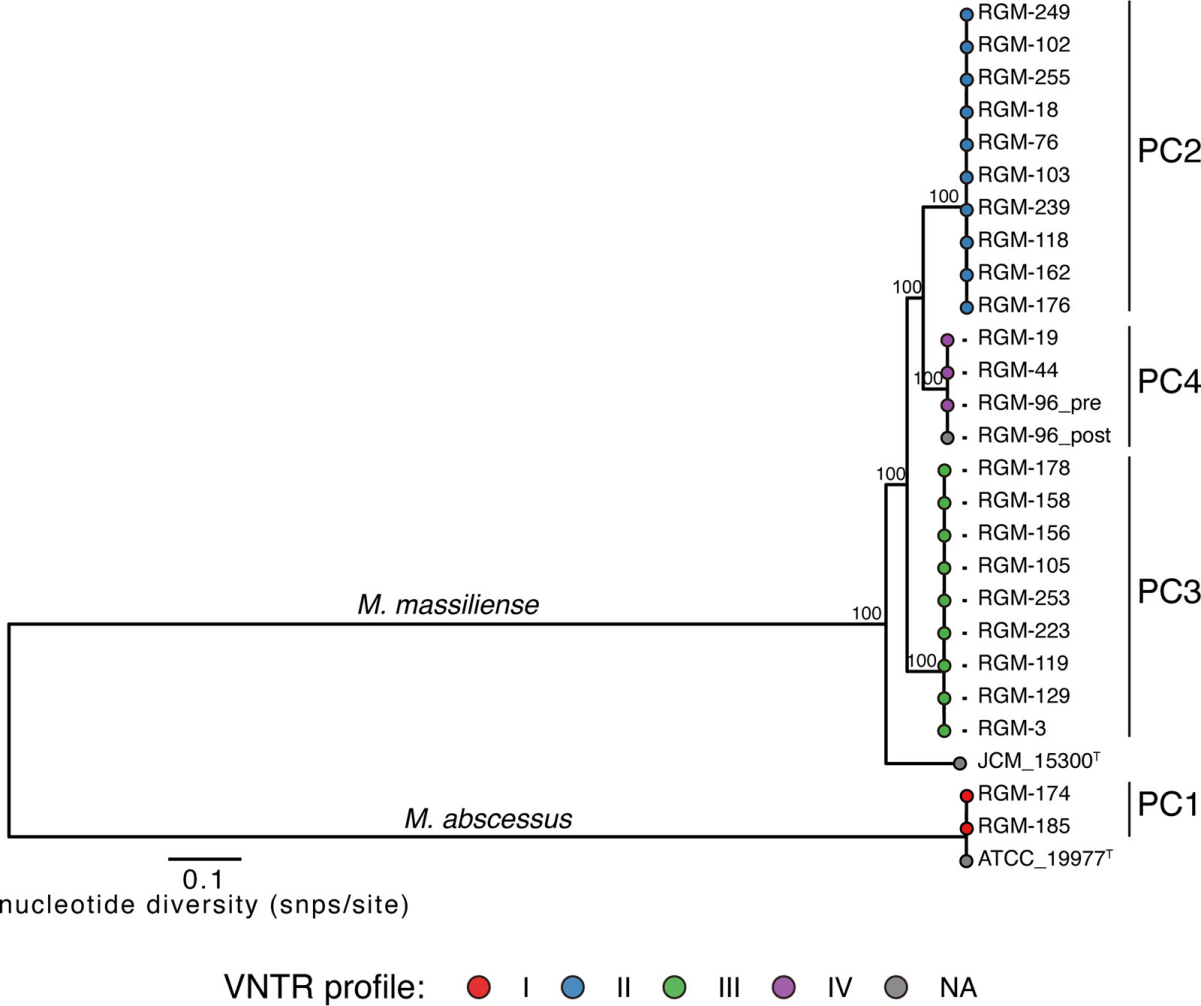
Phylogenetic tree of 25 M. abscessus isolates. The 25 isolates included 24 isolates that composed 4 clusters according to their VNTR profiles and 1 serial isolate obtained from the same patient at a different time point (RGM-96_post). The complete genome sequence of M. abscessus subsp. *massiliense* JCM 15300 was used as the reference. Multiple whole-genome alignments containing 113,083 recombination-free variable positions located in the core genome (4,212,890 bp of the M. abscessus subsp. *massiliense* JCM 15300 genome) were performed to estimate a maximum likelihood tree with 1,000 bootstrap replicates. The scale bar represents the number of substitutions per site (SNPs/site) on the respective branch. VNTR, variable-number tandem repeats; SNP, single nucleotide polymorphism; M. abscessus, Mycobacterium abscessus subsp. *abscessus*; *M. massiliense*, Mycobacterium abscessus subsp. *massiliense*; NA, not applicable; PC, phylogenetic clade.

The phylogenetic tree of the 25 isolates generated using the WGS data is shown in Fig. 1. Several phylogenetic clades (PCs) (PCs I to IV) of M. abscessus subsp. *a*bscessus and M. abscessus subsp. *massiliense* with low genetic diversity were identified, all of which were consistent with each of the four VNTR clusters classified in a previous study (3). One M. abscessus subsp. *massiliense* isolate (RGM-96_post) obtained serially from the patient from whom RGM-96_pre was isolated at a different time point was assigned to the same PC IV as RGM-96_pre.

### SNP analysis and clustering analysis.

**FIG 2:**
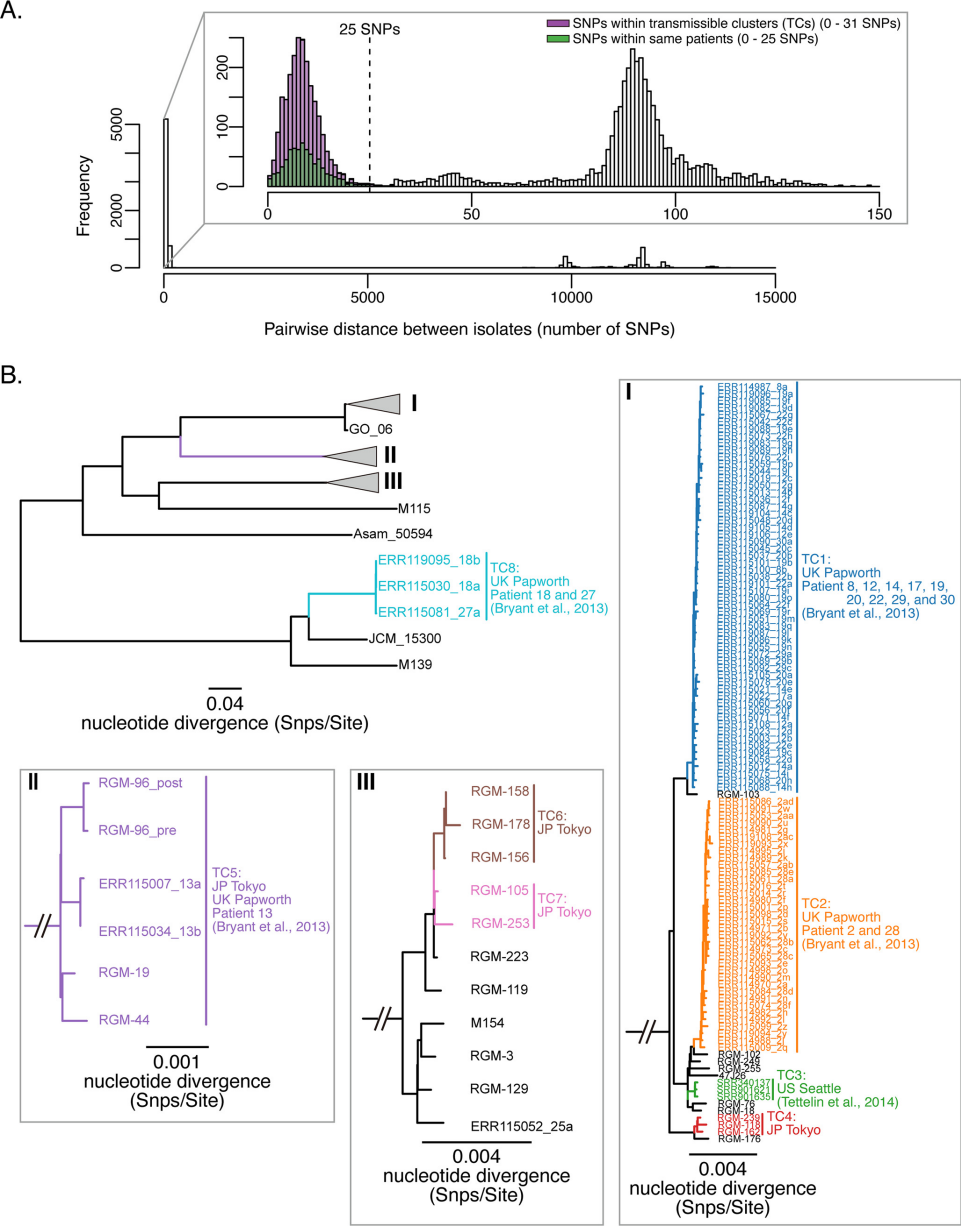
(A) Histogram of pairwise SNP distances among isolates collected at Fukujuji Hospital in Japan, Papworth Hospital in the United Kingdom, and Seattle Hospital in the United States. Whole-genome alignment of a total of 133 M. abscessus subsp. *massiliense* isolates containing 39,364 recombination-free variable positions located in the core genome (4,124,515 bp of the M. abscessus subsp. *massiliense* JCM 15300 genome) was performed to calculate SNP distances among isolates. The maximum SNP distance among isolates consecutively obtained from the same individual was 25, and that between isolates assigned to transmissible clusters (TCs) in the present study was less than 31. (B) Clustering analysis for the identification of possible cross-transmission events between patients. Whole-genome alignment, as described for panel A, was used to estimate a maximum likelihood tree with 1,000 bootstrap replicates. The resulting phylogenetic tree and SNP thresholds (25 SNPs) were used to identify eight TCs using rPinecone. The United Kingdom isolates were assigned to four TCs (TC1, TC2, TC5, and TC8); TC1 and TC2 were consistent with previously reported clusters, and the remaining nonclustered isolates were assigned to TC5 and TC8. All United States isolates were assigned to TC3, and 12 isolates from Fukujuji Hospital were assigned to four clusters (TC4, TC5, TC6, and TC7). TC5 consisted of four isolates from Fukujuji Hospital and two isolates from the United Kingdom. The scale bar represents the number of substitutions per site. SNP, single nucleotide polymorphism; UK, United Kingdom; US, United States; TC, transmissible cluster; *M. massiliense*, Mycobacterium abscessus subsp. *massiliense*; JP, Japan.

The maximum SNP distance among M. abscessus subsp. *massiliense* isolates collected from the same individuals in three hospitals (Fukujuji Hospital in Japan, Papworth Hospital in the United Kingdom, and Seattle Hospital in the United States) was 25 in our analysis (Fig. 2A) (6, 11). First, we investigated the SNP distances among isolates in each PC and identified 15 combinations (17.2%) with less than 25 SNP distances (2 combinations in PC II, 9 in PC III, and 4 in PC IV) (see Fig. S1 in the supplemental material). There were 5 SNP distances between RGM-96_pre and RGM-96_post, which were isolated from the same patient. Next, we identified eight transmissible clusters (TCs) using the SNP distance as a threshold for clonal expansions (Fig. 2B). The United Kingdom isolates were assigned to four TCs (TC1, TC2, TC5, and TC8); the isolates in TC1 and TC2 were identical to those from previously reported nosocomial outbreaks (M. abscessus subsp. *massiliense* clusters 1 and 2), and the remaining nonclustered isolates were assigned to TC5 and TC8 (6). All isolates from a nosocomial outbreak at a CF center in the United States were assigned to TC3 (11), and 12 isolates from Fukujuji Hospital were assigned to four clusters (TC4, TC5, TC6, and TC7). TC5 consisted of four isolates from Fukujuji Hospital and two isolates from the United Kingdom, and the SNP distances between RGM-19 (Fukujuji Hospital) and the 13a and 13b isolates (from the United Kingdom) were 21 and 19, respectively (Fig. S2) (6).

### Epidemiological linkages among and clinical characteristics of the clustered patients.

**FIG 3:**
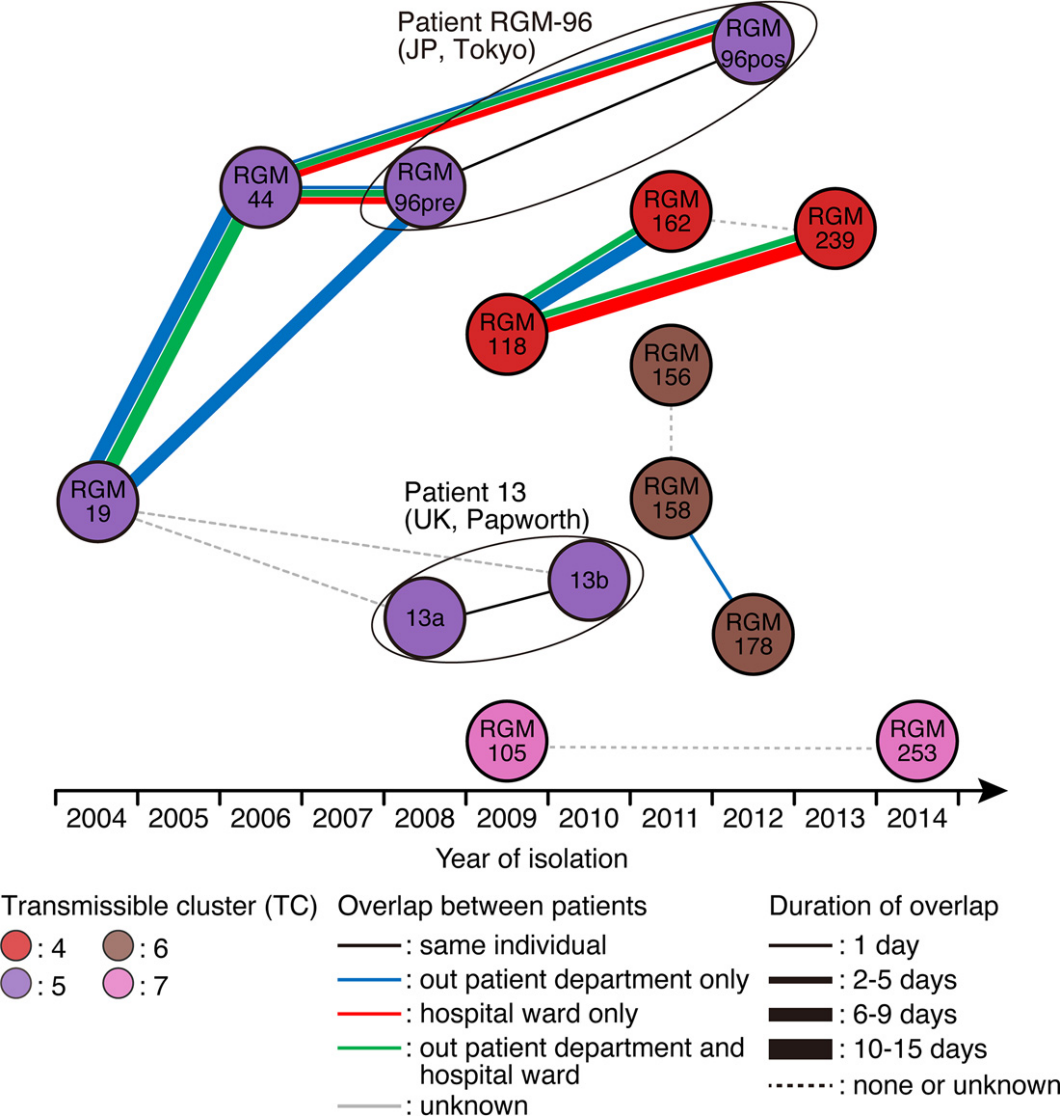
Contact situations among patients in each cluster. The position of each circle indicates the year that the corresponding M. abscessus isolate was obtained from each patient. The circled isolate pairs RGM96pre/RGM96post and 13a/13b were isolated from the same patient. Circles colored with different colors represent each transmissible cluster (TC). TC4 is colored red, TC5 is purple, TC6 is brown, and TC7 is pink. We considered opportunities for cross-transmission within the hospital to have occurred when a patient who once had a positive sputum culture was in the outpatient department or hospital ward or the outpatient department and hospital ward on the same date as another patient, and we counted these instances as overlapping days. The line thickness indicates the number of days the patients were in the hospital on the same date. TC, transmissible cluster; UK, United Kingdom.

The total numbers of overlapping days when the patients were in the outpatient department, hospital ward, and outpatient department-hospital ward on the same date were 6, 8, and 4 days for TC4 and 21, 3, and 13 days for TC5, respectively (Fig. 3). When hospitalized on the same dates, TC4 patients (RGM-118 and RGM-239) were hospitalized on the same floor, and TC5 patients (RGM-44 and RGM-96) were on different floors. Accordingly, patients in TC4 and TC5 shared a total of 18 and 37 days in a close setting, respectively, while TC6 patients had only one outpatient visit on the same date and TC7 patients were not in the hospital at all on the same date. In TC4, after testing positive for acid-fast bacilli (AFB) by sputum culture, patient RGM-118 was in our hospital for the first time on the same date as RGM-162, who was culture-negative. In TC5, a similar situation was observed between RGM-44 (positive) and RGM-96 (negative) (Fig. S2). In contrast, RGM-118 and RGM-239 in TC4, RGM-19, RGM-44, and RGM-96 in TC5, and RGM-158 and RGM-178 in TC6 were culture-positive before their first overlap in our hospital. Although TC5 consisted of both Japanese and United Kingdom-based patients, there was no evidence of contact among them (Fig. 3). Regarding the residences of the Japanese patients assigned to each lineage clade, patients in each cluster resided within a minimum of approximately 13 km (TC6) and a maximum of approximately 20 km (TC7) radius (Fig. S3). There was no specific cluster related to the residences or water supply area. Only two patients were working at the time of sample collection, and workplace-related clusters were not identified. In addition, there were no specific clinical characteristics in each cluster, except for patients in TC4, who showed the same NC-NB radiological patterns and good treatment responses (Table 1).

## DISCUSSION

In the present study, we aimed to clarify the possibility of cross-transmission of M. abscessus among non-CF patients. Using our analysis pipeline, WGS analysis of publicly available isolates from the United Kingdom and the United States showed that the maximum SNP distance within the same individuals was 25, consistent with a previous report (6). Using this SNP distance as a threshold for clonal expansions, we identified four clusters suspected to contain cross-transmissible isolates in our hospital. When investigating epidemiological linkages among our patients, we found that isolates in two clusters were suspected to have undergone direct or indirect transmission. Interestingly, TC5, which contained isolates from Fukujuji Hospital and the United Kingdom, had less than 21 SNP distances; the reason for this is unknown. Although we could not access the hospital or home/work environments, the present study revealed that clonal infections existed even in the non-CF population, and some cases were suspected to be cross-transmissible.

To investigate the possibility of M. abscessus cross-transmission events, Bryant et al. first estimated the SNP distance threshold for isolates using multiple isolates from individual patients (6). Subsequent studies applied the same threshold to their data sets to identify M. abscessus cross-transmission events (12, 13). However, the calculation of SNP distance based on the alignment of clinical isolates to a reference genome is highly dependent on the quality of the data set and the analysis pipeline. Therefore, we evaluated whether our analysis pipeline could investigate the possibility of cross-transmission using our data set containing both isolates obtained from two previous CF center outbreaks in the United Kingdom (6) and the United States (11) and isolates from non-CF patients in this study. Using rPinecone software, in which the SNP threshold was used to define sublineages (19), we thoroughly investigated clusters associated with possible cross-transmission events. Our analysis confirmed that the M. abscessus isolates from the United Kingdom and the United States had classifications similar to those of previously reported clusters (6, 11), and the 12 M. abscessus isolates from our hospital constituted clusters. Based on these findings, we clustered cross-transmissible isolates in CF patients using the present method and found that some isolates clustered similarly in non-CF patients using integrated SNP breakpoints.

We further investigated the epidemiological linkages of each cluster to determine whether M. abscessus non-CF patients who were clustered in the present study had direct or indirect contact with an infectious source. Previous reports suggested that hospitals might be transmission sites based on overlapping outpatient and inpatient dates among patients and network analysis results (6, 20). Considering that our hospital is a four-story medium-size hospital with 205 respiratory beds, direct or indirect contact between outpatients and inpatients or between inpatients on different floors could occur, and opportunities for cross-transmission between patients in clusters TC4 and TC5 within Fukujuji Hospital might have occurred, but these opportunities were limited between patients in clusters TC6 and TC7. In some cases, both patients already had positive sputum cultures when they were admitted to our hospital for the first time on the same date. Therefore, clarifying the transmission route based on only the contact conditions within the hospital was difficult. It is also difficult to identify a potential environmental source of transmission (12). M. abscessus exists in domestic water after sterilization and can be transmitted through aerosols in the bathroom (21, 22). However, relationships between specific clusters and patients’ residences, water supply areas, or workplaces were not observed.

Interestingly, TC5, which contained the WGS data of isolates from Fukujuji Hospital in Japan and Papworth Hospital in the United Kingdom, harbored less than 21 SNP distances. However, these United Kingdom isolates were not M. abscessus subsp. *massiliense* DCCs (14), which have been suspected to circulate among CF patients, and there was no opportunity for close contact between Japanese and United Kingdom patients. This might suggest the existence of successful M. abscessus clones worldwide; however, the global transmission of M. abscessus among non-CF patients has seldom been reported (16). To validate the present data, WGS studies analyzing larger samples in larger non-CF populations are needed, and prospective studies investigating detailed contact opportunities are warranted to further clarify the possibility of cross-transmission.

Our study has several limitations. First, because the present study had a retrospective design and was conducted in a single facility, the number of investigated patients was small, and detailed contact conditions could not be confirmed. Second, although Bryant et al. determined the SNP distance threshold using multiple isolates from individual CF patients (6), SNP distances between multiple isolates from an individual patient were investigated in only one patient in Fukujuji Hospital. Therefore, it was not sufficiently clear whether the threshold used in our pipeline is an appropriate threshold for non-CF patients. Considering that the genomic diversity of M. abscessus isolates from non-CF patients was greater than that of isolates from CF patients (23), the threshold used in our pipeline may be restricted to CF patients. To determine the appropriate threshold for non-CF patients, the SNP distances between multiple isolates from individual non-CF patients should be investigated. Third, WGS was performed mainly on isolates that formed VNTR clusters. In a previous study conducted in 2014 (3), in which we did not implement WGS, we clarified the relationships between VNTR and clinical characteristics by referring to published data (9). Unexpectedly, we found several identical VNTR genotypes in the study. We presumed that isolates without matching VNTR profiles would also not be matched in WGS (24). Thus, this study focused on isolates that formed VNTR clusters; however, less than half of the examined isolates formed transmissible clusters according to SNP analysis, suggesting that the association between VNTR and WGS clusters was not strong. If WGS was performed on all isolates, more transmissible clusters might be identified, and accordingly, direct WGS analysis should be performed in similar studies. Fourth, analysis of core genomic regions alone might not be sufficient. Recently, possible contributions of the accessory genome, regions acquired by horizontal gene transfer, have been indicated (25, 26). We therefore consider that future research, including analyses of accessory genome regions, is warranted. Fifth, we did not obtain and examine isolates from nosocomial and environmental sources that could be potential sources of infection.

In conclusion, closely related isolates were detected in different patients, even in areas with few CF patients, suggesting the possibility of M. abscessus cross-transmission among non-CF patients. However, the transmission route remains unclear, and further studies are needed.

## MATERIALS AND METHODS

### Study patients and microbiological data collection.

This study analyzed 104 M. abscessus isolates stored at Fukujuji Hospital, Japan Anti-Tuberculosis Association, which is a specialized facility for respiratory diseases (205 exclusive beds) located in northwestern Tokyo, Japan, between September 2004 and December 2014. Basically, the first available positive culture from each patient was stored. Of the 104 M. abscessus isolates, 57 isolates were identified as subsp. *abscessus*, 45 as subsp. *massiliense*, and 2 as subsp. *bolletii* using multiplex PCR and *rpoB* gene sequencing as previously described (27, 28). The methods for the MIC, colony morphology evaluation, and VNTR genotyping are described in the supplemental material (3, 9, 29). We performed WGS on isolates that formed VNTR clusters.

### Whole-genome sequencing.

According to a previous report, M. abscessus DNA was extracted (30). A WGS library was constructed using a QIAseq FX DNA library kit (Qiagen, Hilden, Germany). Sequencing was performed on an Illumina NextSeq 550 platform using the NextSeq reagent kit (300 cycles) with 150-mer paired-end short reads. Raw read data were deposited in the DNA Data Bank of Japan (DDBJ) under BioProject accession number DRA012694.

### Phylogenetic analysis and SNP analysis.

Phylogenetic analysis was performed using the core genome, and the detailed methods are described in the supplemental material (31–33). To systematically explore cross-transmission events of M. abscessus between patients, we made the following assumption: the SNP distance between clinical isolates from different patients is smaller than the maximum SNP distance between clinical isolates that are consecutively isolated from the same individual. In these cases, we suspected the possibility of cross-transmission via people and the environment and performed epidemiological linkage investigations. To test this assumption, we combined our data set with publicly available WGS data on 109 M. abscessus subsp. *massiliense* clinical isolates that were consecutively isolated from patients involved in previous nosocomial outbreaks in the United Kingdom (6) and the United States (11). Accordingly, M. abscessus subsp. *massiliense* clinical isolates were especially investigated in the present study. Additional raw read data were obtained from the Sequence Read Archive (SRA) and *de novo* assembled using the Shovill pipeline. To assess the assembly quality of the 109 isolates, we calculated the genome fraction (%) of each isolate, which is the total number of bases aligned with the reference genome (M. abscessus subsp. *massiliense* JCM 15300), using QUAST software (34). We included only isolates with genome fractions greater than 85% in the downstream SNP analyses. The whole-genome alignment of 132 M. abscessus subsp. *massiliense* clinical isolates was performed as noted in the supplemental material, and these data were used to calculate the SNP distances among clinical isolates using snp-dists (https://github.com/tseemann/snp-dists). To identify possible cross-transmission events, we used rPinecone software to define sublineages from clonal expansions (19), in which the maximum SNP distance among isolates from the same individuals (25 SNPs; Fig. 2A) and a phylogenetic tree estimated by RAxML software were used as input parameters (33).

### Clinical and epidemiological analyses.

To analyze the clinical characteristics and epidemiologic linkages among patients, we collected data on sex, age, past medical histories, radiological findings, patient residences, work histories, dates of outpatient visits (including visits for outpatient examinations such as blood tests, radiographic examinations, and physiological function tests), and admission periods and wards from the first visit to Fukujuji Hospital through the end of the study. We considered that opportunities for nosocomial cross-transmission could occur if a patient who once had a positive sputum acid-fast bacilli (AFB) culture shared the same space at the same time with another patient in the ward or at the outpatient clinic. We counted those days and investigated the possibility of contact. Radiological findings based on computed tomography were classified into the following disease patterns: fibrocavitary type (FC type), cavitary nodular bronchiectatic type (C-NB type), noncavitary nodular bronchiectatic type (NC-NB type), and unclassified type (35). Regarding treatment outcomes, we referred to the Nontuberculous Mycobacteria Network European Trial Group (NTM-NET) consensus statement (36). We defined “treatment success” as more than three consecutive negative cultures collected at least 4 weeks apart and maintenance of negative culture conversion until the end of the study, which was similar to the microbiological cure criteria (36).

The study protocol was approved by the Fukujuji Hospital Institutional Review Board (protocol number 21024), and informed consent was waived because of the retrospective nature of the analysis.

### Data availability.

The raw read data of whole-genome sequencing were deposited in the DNA Data Bank of Japan (DDBJ) under BioProject accession number DRA012694.
