# Supplementary material for: Potential Cross-Transmission of Mycobacterium abscessus among Non-Cystic Fibrosis Patients at a Tertiary Hospital in Japan
Source: Microbiol Spectr. 2022 May 10;10(3):e00097-22. doi: 10.1128/spectrum.00097-22 (PMC9241747; doi:10.1128/spectrum.00097-22)
Supplement: SUPPLEMENTAL FILE 1 — Supplemental material. Download spectrum.00097-22-s001.pdf, PDF file, 0.9 MB [file spectrum.00097-22-s001.pdf]

Supplementary Material

Potential Cross-Transmission of *Mycobacterium abscessus* among Non-Cystic Fibrosis Patients at a Tertiary Hospital in Japan

Keiji Fujiwara, Mitsunori Yoshida, Yoshiro Murase, Akio Aono, Koji Furuuchi, Yoshiaki Tanaka, Ken Ohta, Manabu Ato, Satoshi Mitarai, Kozo Morimoto

Materials and methods

Analysis of isolates

The minimum inhibitory concentrations (MICs) of clarithromycin and amikacin were measured by the broth microdilution method according to the clinical laboratory standards institute M24 3rd ed. (1). The MICs were evaluated at day 5, but that of clarithromycin was finally evaluated on day 14 because of delayed induction of the *erm*(41) gene. Colony morphology was classified into rough, smooth, mixed, or intermediate (2). With reference to a previous article (3), 9 variable number of tandem repeats (VNTR) loci of *Mycobacterium abscessus* subsp. *abscessus* (Mab-1, 4, 7, 9, 14, 18, 23, 24, and 28) and 10 VNTR loci of *Mycobacterium abscessus* subsp. *massiliense* (Mab-1, 4, 7, 11, 14, 18, 21, 24, 28, and 29) were selected for VNTR genotyping of *Mycobacterium abscessus*.

Phylogenetic analysis

The whole-genome sequencing read data of each isolate were *de novo* assembled into contigs by the Shovill pipeline (https://github.com/tseemann/shovill). The number of contigs, raw coverage, and the N50 value of each isolate are listed in Table S1. We performed pairwise genome alignment of the reference genome (*Mycobacterium*

abscessus subsp. *massiliense* JCM 15300) with one of the 25 isolates and *Mycobacterium abscessus* subsp. *abscessus* ATCC 19977 using MUMmer ver. 3.2.3 (4) to identify genomic regions conserved among all clinical isolates (core-genome) and the SNP sites located within these regions. We then combined all alignments into multiple whole-genome alignments, in which each position corresponded to that of the reference genome, using custom Perl scripts. This alignment was subjected to inference of recombination sites using Gubbins and detected recombinogenic sites were removed from the alignment (5). The resulting alignment was used to reconstruct a maximum-likelihood tree using the RAxML ver. 8.2.12 (6) with the General Time Reversible (GTR)-GAMMA substitution model and 1000 bootstrap replicates.

References

1. Brown-Elliott BA, Woods GL. 2019. Antimycobacterial Susceptibility Testing of Nontuberculous Mycobacteria. J Clin Microbiol 57.
2. Morimoto K, Nakagawa T, Asami T, Morino E, Fujiwara H, Hase I, Tsujimoto Y, Izumi K, Hayashi Y, Matsuda S, Murase Y, Yano R, Takasaki J, Betsuyaku T, Aono A, Goto H, Nishimura T, Sasaki Y, Hoshino Y, Kurashima A, Ato M, Ogawa K, Hasegawa N, Mitarai S. 2018. Clinico-microbiological analysis of 121 patients with pulmonary Mycobacteroides abscessus complex disease in Japan - An NTM-JRC study with RIT. Respir Med 145:14-20.
3. Shin SJ, Choi GE, Cho SN, Woo SY, Jeong BH, Jeon K, Koh WJ. 2013. Mycobacterial genotypes are associated with clinical manifestation and progression of lung disease caused by Mycobacterium abscessus and Mycobacterium massiliense. Clin Infect Dis 57:32-9.
4. Kurtz S, Phillippy A, Delcher AL, Smoot M, Shumway M, Antonescu C, Salzberg SL. 2004. Versatile and open software for comparing large genomes. Genome Biol 5:R12.
5. Croucher NJ, Page AJ, Connor TR, Delaney AJ, Keane JA, Bentley SD, Parkhill J, Harris SR. 2015. Rapid phylogenetic analysis of large samples of recombinant

bacterial whole genome sequences using Gubbins. Nucleic Acids Res 43:e15.

6. Stamatakis A. 2006. RAxML-VI-HPC: maximum likelihood-based phylogenetic analyses with thousands of taxa and mixed models. Bioinformatics 22:2688-90.

A. PCII

	RGM-103	RGM-102	RGM-249	RGM-255	RGM-76	RGM-18	RGM-239	RGM-118	RGM-162	RGM-176
RGM-103										
RGM-102	108									
RGM-249	99	53								
RGM-255	118	76	59							
RGM-76	112	72	63	82						
RGM-18	99	61	50	65	44					
RGM-239	109	120	114	128	125	114				
RGM-118	117	127	121	135	131	120	21			
RGM-162	112	122	116	131	126	115	16	27		
RGM-176	124	135	129	144	140	128	37	48	41	

B. PCIII

	RGM-178	RGM-158	RGM-156	RGM-223	RGM-105	RGM-253	RGM-119	RGM-3	RGM-129
RGM-178									
RGM-158	18								
RGM-156	20	4							
RGM-223	35	20	18						
RGM-105	36	21	19	9					
RGM-253	40	38	36	26	25				
RGM-119	61	46	44	32	33	52			
RGM-3	113	93	91	77	78	98	87		
RGM-129	102	86	84	71	72	91	84	39	

C. PCIV

	RGM-96_pre	RGM-96_post	RGM-44	RGM-19
RGM-96_pre				
RGM-96_post	5			
RGM-44	30	31		
RGM-19	23	24	21	

Fig. S1. SNP distances among the isolates in each phylogenetic clade (PC) (PCs II–IV). The numbers in the boxes represent the SNP distances between isolates shown on the x-axis and y-axis. Regarding RGM numbers, isolates assigned to transmissible clusters (TCs) are noted in a color other than black. TC4 is colored red, TC5 is purple, TC6 is brown, and TC7 is pink. A total of fifteen combinations with SNP distances less than 25 among isolates were identified (two combinations in PC II, nine in PC III, and four in PC IV). SNP, single nucleotide polymorphism.

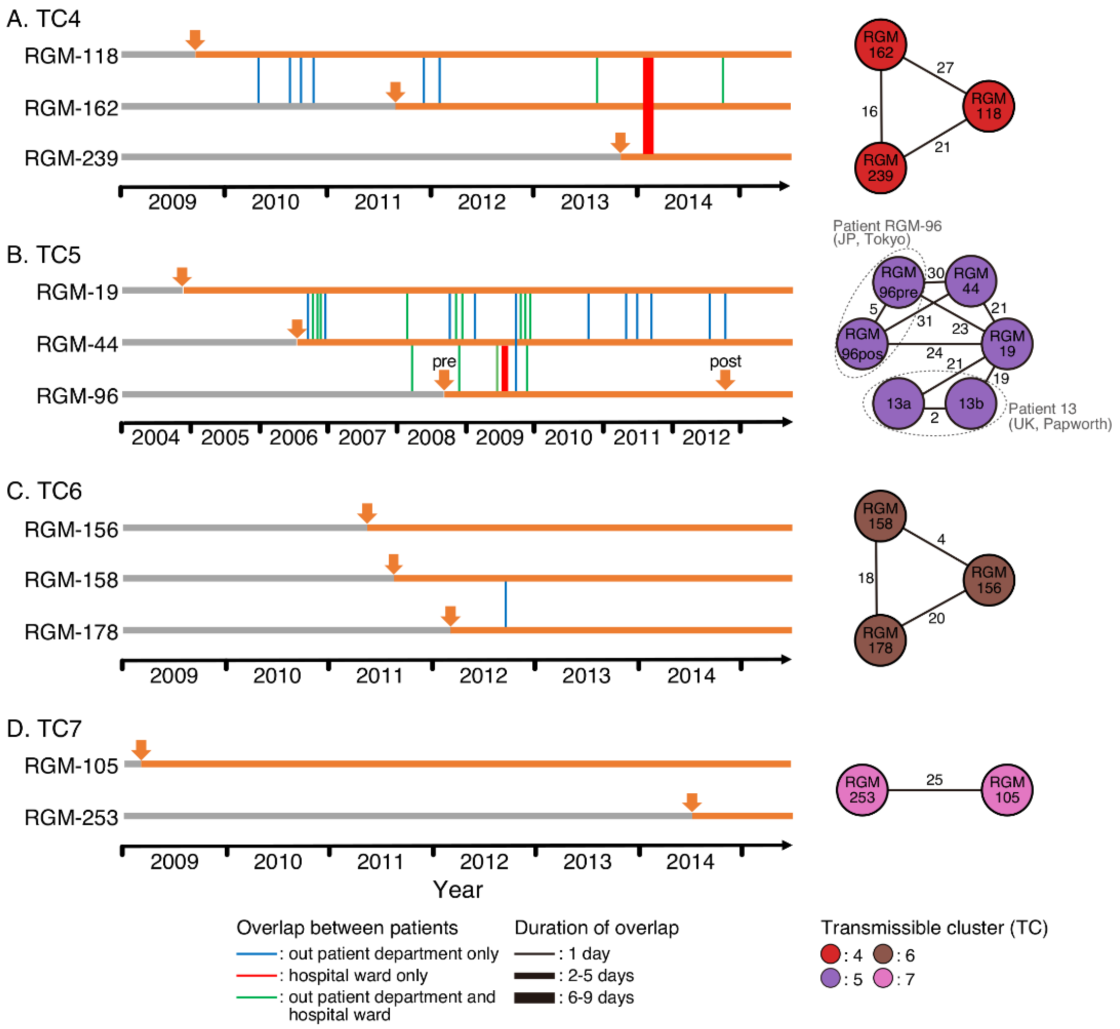

Fig. S2. Opportunities for cross-transmission within a hospital. Timelines for individual patients in transmissible cluster (TC) cluster 4 (A), TC cluster 5 (B), TC cluster 6 (C), and TC cluster 7 (D) are shown. The arrows indicate when *Mycobacterium abscessus* isolate was obtained from each patient for the first time (excluding RGM-96 post). When overlaps occur between patients, the timelines for each patient are connected by blue (outpatient department), red (hospital ward), or green bars (outpatient department and hospital ward). In addition, circles colored with different colors represent each

transmissible cluster (TC). TC4 is colored red, TC5 is purple, TC6 is brown, and TC7 is pink. The numbers beside the lines connecting the circles represent the SNP distances between each isolate. SNP, single nucleotide polymorphism.

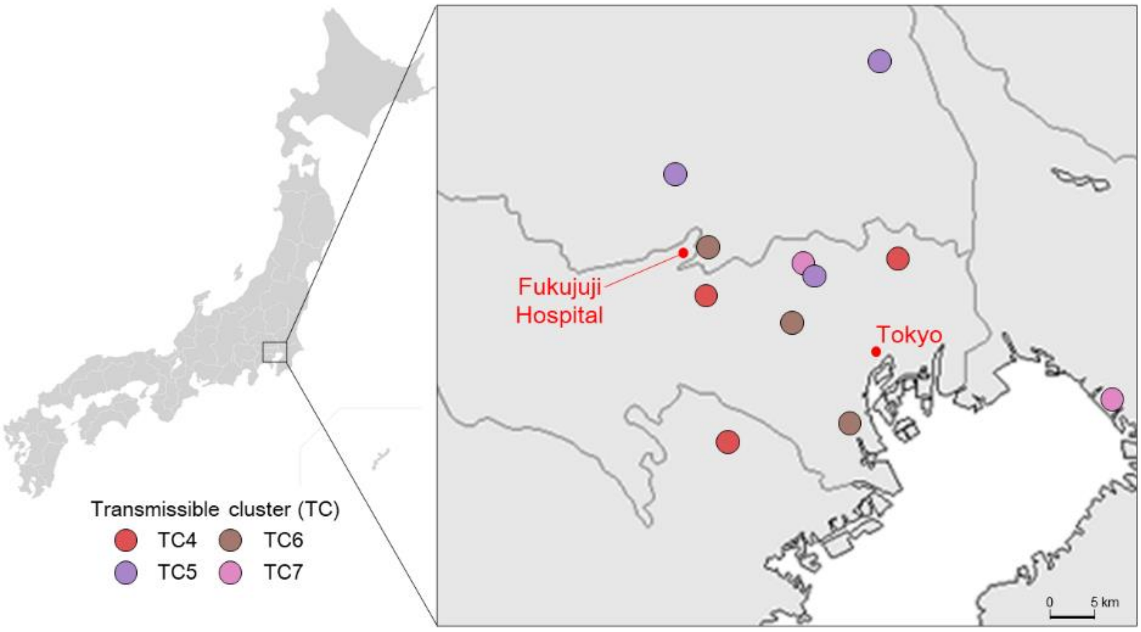

Fig. S3. Patients' residences are plotted as colored circles on the map for each transmissible cluster (TC). TC4 is colored red, TC5 is purple, TC6 is brown, and TC7 is pink. The lines on the map are prefectural boundaries. Water supply services are principally provided on a prefectural basis, and each prefecture has a different water supply area. No transmissible clusters of patients living in the same prefecture were identified.

Table S1. Genomic features of *Mycobacterium abscessus*

Isolate	Contigs	Total length	GC content (%)	N50	Coverage
RGM-3	33	5178732	64.1	427627	164
RGM-18	29	4938127	64.2	758444	240
RGM-19	99	5397016	64.2	153123	237
RGM-44	83	5020364	64.3	162700	163
RGM-76	13	4860439	64.2	678829	185
RGM-96 (pre)	93	5009787	64.3	117335	132
RGM-96 (post)	99	5010753	64.3	101100	126
RGM-102	12	4878627	64.1	674527	205
RGM-103	31	5129256	64.1	435601	167
RGM-105	24	5134092	64.2	388845	182
RGM-118	8	4764922	64.3	2571743	179
RGM-119	21	5080706	64.2	745433	200
RGM-129	37	5085847	64.1	387582	171
RGM-156	21	5107571	64.2	1043114	204
RGM-158	21	5107053	64.2	1043114	257
RGM-162	11	4869155	64.2	1013970	231
RGM-174	364	5645706	64.0	65204	150
RGM-176	15	4803440	64.3	503700	222
RGM-178	169	5595232	64.0	139612	266
RGM-185	36	5185214	64.1	651148	237
RGM-223	36	5187232	64.2	416024	90
RGM-239	21	4952044	64.2	769271	137
RGM-249	30	5041122	64.1	502031	213
RGM-253	112	5232923	64.1	259342	226
RGM-255	26	4854471	64.1	588023	189
